# Molecular Subtyping and Outlier Detection in Human Disease Using the Paraclique Algorithm

**DOI:** 10.3390/a14020063

**Published:** 2021-02-19

**Authors:** Ronald D. Hagan, Michael A. Langston

**Affiliations:** Department of Electrical Engineering and Computer Science, University of Tennessee, Knoxville, TN 37996, USA;

**Keywords:** molecular subtyping, outlier detection, paraclique algorithm, transcriptomic data

## Abstract

Recent discoveries of distinct molecular subtypes have led to remarkable advances in treatment for a variety of diseases. While subtyping via unsupervised clustering has received a great deal of interest, most methods rely on basic statistical or machine learning methods. At the same time, techniques based on graph clustering, particularly clique-based strategies, have been successfully used to identify disease biomarkers and gene networks. A graph theoretical approach based on the paraclique algorithm is described that can easily be employed to identify putative disease subtypes and serve as an aid in outlier detection as well. The feasibility and potential effectiveness of this method is demonstrated on publicly available gene co-expression data derived from patient samples covering twelve different disease families.

## 1. Introduction

It has long been established that many disease families exhibit a wide range of heterogeneity. This is especially true in cancer. Lung cancers, for example, fall into two overall types based on histological characteristics: small cell lung cancer (SCLC) and non-small cell lung cancer (NSCLC). Although histological classification remains crucial, significant advances in the treatment of NSCLC over the last decade have centered around the development of therapies targeting subtypes at the molecular level, such as those defined by genetic mutations [1]. In particular, therapies targeting alterations in the epidermal growth factor receptor (EGFR) and anaplastic lymphoma kinase (ALK) genes have produced dramatic improvements in outcomes for patients in the underlying subgroups [2,3]. In addition to providing new paths for treatment, advances in molecular subtyping allow practitioners to avoid needless high-risk therapies. For example, studies have identified transcriptomic signatures for chemo-resistance in both acute myeloid leukemia and breast cancer [4,5]. Recent research has made positive steps towards targeted care for a variety of diseases, including Asthma, Alzheimer’s, and Crohn’s disease [6–8]. A key development driving these advances is the successful identification of molecular subtypes.

Given the potential impact of disease subtype identification, it is not surprising therefore, that the search for effective clustering methods has become an intense area of interest. Traditional approaches, such as *k*-means and hierarchical clustering, have long been used to identify sets of genes or samples that exhibit similar expression patterns [9–11]. Machine learning techniques based on neural networks have been investigated as well [12–14]. Latent variable and mixture models have also been used [15–17]. Meanwhile, a graph theoretical approach is to model a set of genes or samples as vertices in a graph, with edges connecting them based on thresholding some similarity metric. A systematic comparison of clustering methods over well-annotated *S*. *cerevisiae* (baker’s yeast) gene co-expression data can be found in [18], where it was shown that clique-centric graph theoretical algorithms generally outperform other approaches. Moreover, the top-down paraclique algorithm (see Supplementary Materials) introduced in [19] was found to possess considerable computational advantages over other clique-based tools. Maximal clique [20], for example, is output bound, while k-clique communities [21] are hobbled by bottom-up inefficiencies. Paraclique has seen prior application in transcriptomics [22], proteomics [23], epigenetics [24] and the exposome [25], as well as in the study of specific diseases including lung cancer [26], diabetes [27], allergic rhinitis [28] and community-acquired pneumonia [29], and even in investigations of the effects of radiation on living organisms [30]. Nevertheless, to the best of our knowledge, this paper represents the first attempt to gauge paraclique’s potential merit in the context of molecular disease subtyping.

To address this gap, we describe an initial study of putative subtypes based on molecular signatures using the paraclique method. Our technique is general and applies easily to other types of data such as protein interaction, metabolite abundance, or DNA methylation profiles, but we focus our experimentation on gene co-expression data thanks to its relative quality and ubiquity. In addition to subtype discovery, we will show how our techniques can be used to help pinpoint potential outliers, providing an automatic means for the identification of suspected data collection errors such as mislabeled samples or misdiagnosed patients. We hasten to observe, however, that biological variation can be inscrutable, inconsistent, and unpredictable. No method is therefore likely to be extraordinarily accurate. We will address this and related issues in the sequel.

This paper is organized as follows. In the next section, we provide a brief review of the paraclique algorithm and discuss details of our workflow for subtyping in gene co-expression data. In Section 3, we outline our testing procedures, provide GO enrichment results that indicate functional biological relevance of the subtypes we identify, and describe additional testing with labeled data over known subtypes that demonstrate the fidelity of further stratification using this approach. In Section 4, we consider outlier detection and discuss how methods such as these can help address this problem. In a final section, we summarize results, place them in context, and consider avenues for future work. A preliminary version of a portion of this paper was presented at the International Workshop on Biological Knowledge Discovery from Big Data, held in Linz, Austria, in August, 2019.

## 2. Methodology

**Figure 1.**
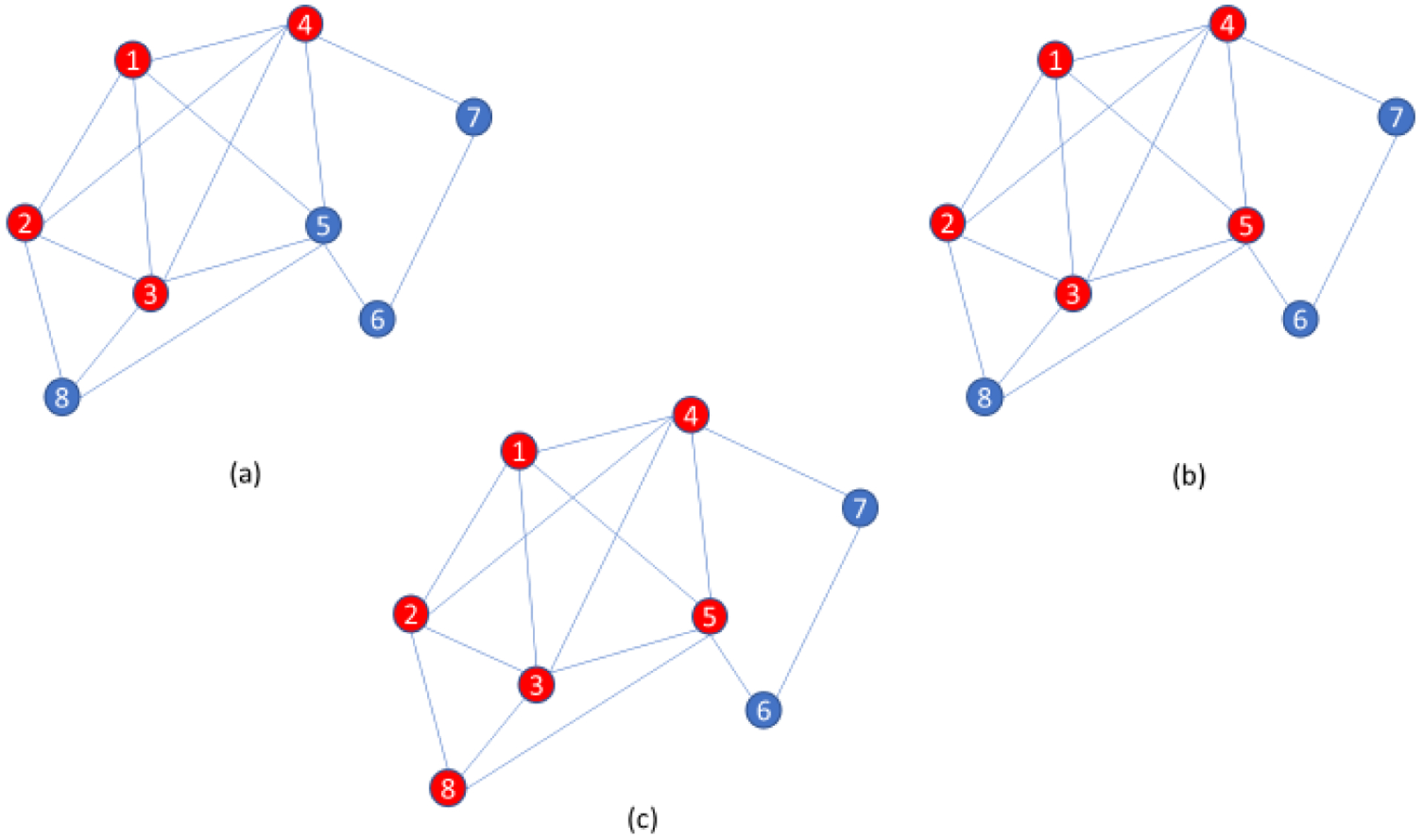
An illustration of the paraclique algorithm with glom term *g* = 2. **(a)** Starting with a maximum clique of size 4 as shown by red vertices, **(b)** paraclique first gloms onto vertex 5, **(c)** and then it glams onto vertex 8 to form a paraclique of size 6.

Clique-centric methods have long been used in a wide variety of applications [31]. On real and noisy data, however, clique finders may be inherently prone to high false negative rates. Indeed, an entire clique may be missed if even a single edge is lost. Thus, the paraclique algorithm is an effort to ameliorate difficulties posed by noise. Its essential strategy is first to isolate a maximum clique, and then expand it by glomming onto any new vertex that is adjacent to all but some predefined number of vertices already in this clique. This number is termed the glom term, *g*. An illustration of paraclique construction with *g* = *2* is provided in Figure 1. Paraclique details and a thorough discussion of clique selection, edge weights, densities, and other important algorithmic features can be found in [32,33]. Web-based versions of paraclique and related tools are available to the community via GrAPPA [34].

In this effort, we were mainly concerned with case-control transcriptomic data, for which we applied an initial filtering step to limit the effects of confounding factors. False discovery rate adjusted *p*-values for the differential expression of genes between case data and control data were calculated using the Benjamini-Hochberg method [35] accessed via the Entropy Explorer R package [36]. Only those genes with *p*-values less than or equal to 0.1 were retained. The motivation for such a filter was to restrict attention to genes of potential interest in the differential diagnosis of disease. After all, we wanted to concentrate on potential disease subtypes and not be distracted by irrelevant subgroups such as age, ethnicity, or hair color. Once filtering was complete, we focused our attention only on case data, and reversed the roles of variables and correlations. We therefore calculated pairwise Pearson correlation coefficients between samples (not genes), and across their corresponding lists of expression levels (not patients). We then thresholded the resultant correlation matrix using spectral methods as in [37] and constructed an unweighted graph with vertices representing samples and edges between highly correlated sample pairs. Once this graph had been created, we invoked the paraclique algorithm to extract dense, noise resilient subgraphs. Thus, each such subgraph represented a putative subtype or outlier.

For consistency, and because this work mainly represents a proof of concept, we set the glom term to *g* = 1 throughout this effort. Depending on the data under study, however, crisper results may naturally be anticipated with fine tuning. In [27], for example, a glom term of *g* = 5 was found to produce superior ontological enrichments when studying non-obese diabetic mice as a model of type 1 diabetes mellitus.

## 3. Experimental Results

### 3.1. Discussion

We applied this novel analytical approach to a dozen sets of publicly available gene co-expression data obtained from the Gene Expression Omnibus (GEO). These data were selected because they provide a wide cross-section of human disease, and because each has both a case and a control group for the aforementioned filtering task. Table 1 provides an overview of the datasets we studied.

Our investigation into the effectiveness of this proposed new methodology was focused on two guiding questions: (1) Are these tools capable of reliably and robustly identifying putative subtypes? (2) Are these subtypes appropriate to the associated disease as supported by biological evidence from clinical, published, analytical or other orthogonal information source(s)?

The answer to the first question seems to be an unequivocal yes. As summarized in Table 2, our methods decomposed raw data into putative subtypes in ten of our datasets. In the case of asthma, for example, every patient sample fell into some paraclique. In other cases, patients were sometimes left unclassified, which is hardly surprising given the limitations on dataset sizes coupled with possible extremes in disease as well as sample heterogeneity. Only for Parkinson’s disease and type 2 diabetes were no subtypes identified. It is probably no coincidence then that these two diseases also have by far the smallest datasets, especially in light of clinical subtyping evidence to the contrary [38,39].

The second question is considerably more difficult to answer because it depends on the availability of alternate, non-transcriptomic data sources. We therefore followed a two-pronged approach in putative subtype comparisons. First, we calculated GO enrichments and their associated *p*-values for the top 100 differentially expressed genes in each paraclique. These results and their corresponding GO categories are summarized in Table 3. In every case, we found statistical evidence for biological significance among the genes separating samples into subgroups, with enrichment *p*-values ranging from 1.1 × 10^−4^ for asthma to 4.92 × 10^−46^ for prostate cancer. Next, we performed a literature search to check the top scoring genes for involvement in known subtypes. As such, this is at best a hit-or-miss proposition, and one depending for each disease on whether the research community has studied subtyping issues, found results, and published them in venues that we were able to search. Despite these obstacles, however, we found strong evidence in print to support our putative subtype decompositions for four of the diseases we studied. These are asthma, breast cancer, chronic lymphocytic leukemia, and colorectal cancer.

### 3.2. A Search for Unrecognized Subtypes

#### 3.2.1. Asthma

The incidence of asthma in the U.S. has been on the rise for two decades. It is currently estimated that nearly one in ten children under 18 are asthmatic. The risk for some groups is based largely on ethnicity (particularly African American and Puerto Rican), with incidence among those with lower socioeconomic status rising as high as one in six [40].

GEO series GSE4302 data were derived from the Affymetrix Human Genome U133 Plus 2.0 Array, and are designed to identify genes associated with response to corticosteroid treatment in asthmatics [41]. They consist of transcriptomic data taken from the epithelial airway brushings of 42 asthmatics, 28 healthy subjects and 16 smokers. To avoid potential confounds, we discarded data taken from smokers and used only the healthy subjects as controls.

Filtering reduced the number of probes from 54,676 to 2322. Our method produced three paracliques with respective sizes 31, 8 and 3 that were stable until they began to merge as the threshold was lowered below 0.93. The 100 most differentially expressed genes across the two larger putative subtypes included CLCA1, periostin, and ovalbumin, which are all known to serve as markers of a Th2-high endotype of asthma [42].

#### 3.2.2. Breast Cancer

Genetic factors have long been known to play a significant role in breast cancer. Studies have shown that in families with at least four breast cancer cases, most can be linked to mutations in either BRCA1 or BRCA2 genes [43,44]. Moreover, breast cancer has a variety of known subtypes that significantly impact prognosis and treatment. For example, tumors negative for estrogen receptors, progesterone receptors, and the expression of HER2 are indicative of triple-negative breast cancer, a subtype identified with higher risk of recurrence and a five-year mortality rate [45].

GEO series GSE10810 data were also derived from the Affymetrix Human Genome U133 Plus 2.0 Array, although values for only 18,382 probes were provided. This study was designed to investigate links between gene co-expression and phenotypic breast cancer differences [46], and contains data for 31 tumor samples and 27 healthy tissues.

Filtering reduced the number of probes to 11,531. Our tools produced two paracliques of size 22 and 5 that persisted to a threshold of 0.8 and left four tumor samples unclassified. The 100 most differentially expressed genes between these putative subtypes include SLC39A6, S100a4, AGR3, Cd24, and epcam, all of which have been reported in the literature as biomarkers for distinct breast cancer phenotypes [47–51].

#### 3.2.3. Chronic Lymphocytic Leukemia

Chronic lymphocytic leukemia is one of the most common types of leukemia, with pathogenesis characterized by an overproduction of neoplastic B cells in the blood-stream. The current median age at diagnosis is 65, with males affected more often than females [52]. Chronic lymphocytic leukemia typically presents with a slow progression in which patients are able to enjoy a more or less normal life expectancy. In some cases, however, chronic lymphocytic leukemia can be aggressive, with death occurring less than five years after the onset of symptoms.

GEO series GSE8835 data were instead derived from the Affymetrix Human Genome U133A Array with 22,283 probes, and were designed to study the effects of chronic lymphocytic leukemia on T cells in peripheral blood [53]. The study comprised 24 CD4 cell samples from chronic lymphocytic leukemia patients and 12 CD4 cell samples from healthy, age-matched donors.

Filtering reduced the number of probes to 1338. At a threshold of 0.8, our tools produced two paracliques of size 4 and 18, leaving two samples unclassified. The most differentially expressed genes across these two putative subtypes included ZAP-70, previously identified as the best discriminator of Ig-mutated and Ig-unmutated chronic lymphocytic leukemia [54].

#### 3.2.4. Colorectal Cancer

The incidence of colorectal cancer has been in decline since the mid 1980s [55]. Despite this significant drop in prevalence, it still accounts for both the third highest number of new cases of cancer, and the third highest number of cancer deaths each year [56]. As with breast cancer, there are known hereditary links to this disease. For example, a mutation of the gene APC is responsible for two syndromes, Familial Adenomatous Polyposis and Hereditary Nonpolyposis Colorectal Cancer, that each carry a significant increase in the risk of developing colorectal cancer [57].

GEO series GSE9348 data were again derived from the Affymetrix U133 Plus 2 array, and were intended to search for transcriptomic signatures of early stage colorectal cancer that is prone to metastasis [58]. The study contains gene co-expression data that were taken from 70 colorectal cancer patient tumors, as well as tissues from 12 healthy subjects who were matched by age and ethnicity.

Filtering reduced the number of probes from 54,675 to 22,968. At a threshold of 0.87, our tools produced two paracliques of size 63 and 5, covering all but two of the case samples. The list of 100 genes most differentially expressed between these two putative subtypes include Cd24, identified as a prognostic marker for colorectal cancer [59] as well as OLFM4, indicated in as a marker for tumor differentiation and progression [60,61].

### 3.3. Alignment with Previously Known Subtypes

The experimental effort just described suggests that our methods have the potential to identify both known and novel subtypes, as based on biologically relevant genetic signatures. The lack of any widespread established ground truth, however, places a limitation on any in-depth interpretation of these results. In an effort to address this shortcoming, we identified two sets of publicly available data on GEO that include metadata labeling in the form of known subtyping information. These are based on gastric cancer and non-small cell lung cancer. As our intent is to identify and contrast novel subtypes in disease, our metric of interest is patient stratification.

#### 3.3.1. Gastric Cancer

GEO series GSE35809 data, from the Affymetrix Human Genome U133 Plus 2.0 Array, were derived from 70 primary gastric tumors intended for use as a validation set for subtype classifier testing [62–64]. The data contain values for 54,675 probes. Arrays are subdivided into a collection of 29 identified as coming from proliferative tumors, 26 from invasive, and 15 from metabolic.

Filtering was irrelevant, because no healthy tissues were studied that could be used as controls. At a threshold of 0.955, paraclique produced subsets of size 29 and 16 and performed admirably. All but one of the invasive samples it classified were placed in the first paraclique, while all but one of the proliferative samples it classified were placed in the second. Metabolic samples proved only slightly more challenging, with 75% of those classified placed in the first paraclique. See Table 4.

#### 3.3.2. Non-Small Cell Lung Cancer

GEO series GSE10245 data, also from the Affymetrix Human Genome U133 Plus 2.0 Array with 54,675 probes, were derived from 40 adenocarcinoma tumors and 18 squamous cell carcinoma tumors, NSCLC’s two most prevalent subtypes. These data were intended to provide a basis for studying co-expression differences between these two cancers [65].

Filtering was again irrelevant. Paraclique performed quite well on these data too. At a threshold of 0.94, it produced three subsets of size 26, 12 and 8. Roughly 74% of the adenocarcinoma samples were placed in the first paraclique while the second contained none, and 80% of the squamous cell carcinoma samples were placed in the second paraclique while the third had none. Again, see Table 4.

#### 3.3.3. Comparison with Other Methods

We sought to compare this basic and untuned version of the paraclique algorithm with well-known strategies such as k-means and hierarchical clustering, as implemented in core-R through the functions *kmeans* () and *hclust* ().

Results for the k-means method were mixed. It proved extremely successful on the gastric cancer data. There it divided samples into two subsets of size 26 and 44. All but one of the invasive samples were placed in the first cluster, while all of the proliferative and all but one of the metabolic samples were placed in the second. However, k-means failed completely on the non-small cell lung cancer data. Samples were divided into subsets of size 28 and 30, with both the adenocarcinoma and the squamous cell carcinoma samples spread almost evenly across these two clusters. The hierarchical approach was also a rather uneven performer. On the gastric cancer data, it divided samples into two subsets of size 33 and 37. While all of the proliferative samples found their way to the second cluster, the first contained 84% of the invasive and about 73% of the metabolic. On the non-small cell lung cancer data, it divided samples into subsets of size 9 and 49. All the adenocarcinoma samples were admirably grouped in the second cluster, but the squamous cell carcinoma samples were not convincingly stratified at all, with exactly half placed in each cluster. These results are also summarized in Table 4.

As demonstrated by these experiments, the paraclique methodology can provide excellent patient stratification, further motivating the use of graph theoretical methods to differentiate samples based on their underlying genetic signatures. Such stratification is not perfect, of course, nor should we expect it to be given data limitations and biological variability. Moreover, unlike techniques such as k-means and hierarchical clustering, patients are not forced into a cluster under paraclique, as is evidenced by the 25 samples it left unclassified in the gastric cancer data. We suggest therefore that the tools we have described here may be best suited to fast screening tasks, for example, when transcriptomic data are relatively easy to obtain. Once clinical and/or additional forms of data have been collected, histological and other more laborious techniques will likely help provide more comprehensive subtyping of entire patient populations.

## 4. Outlier Detection

**Figure 2.**
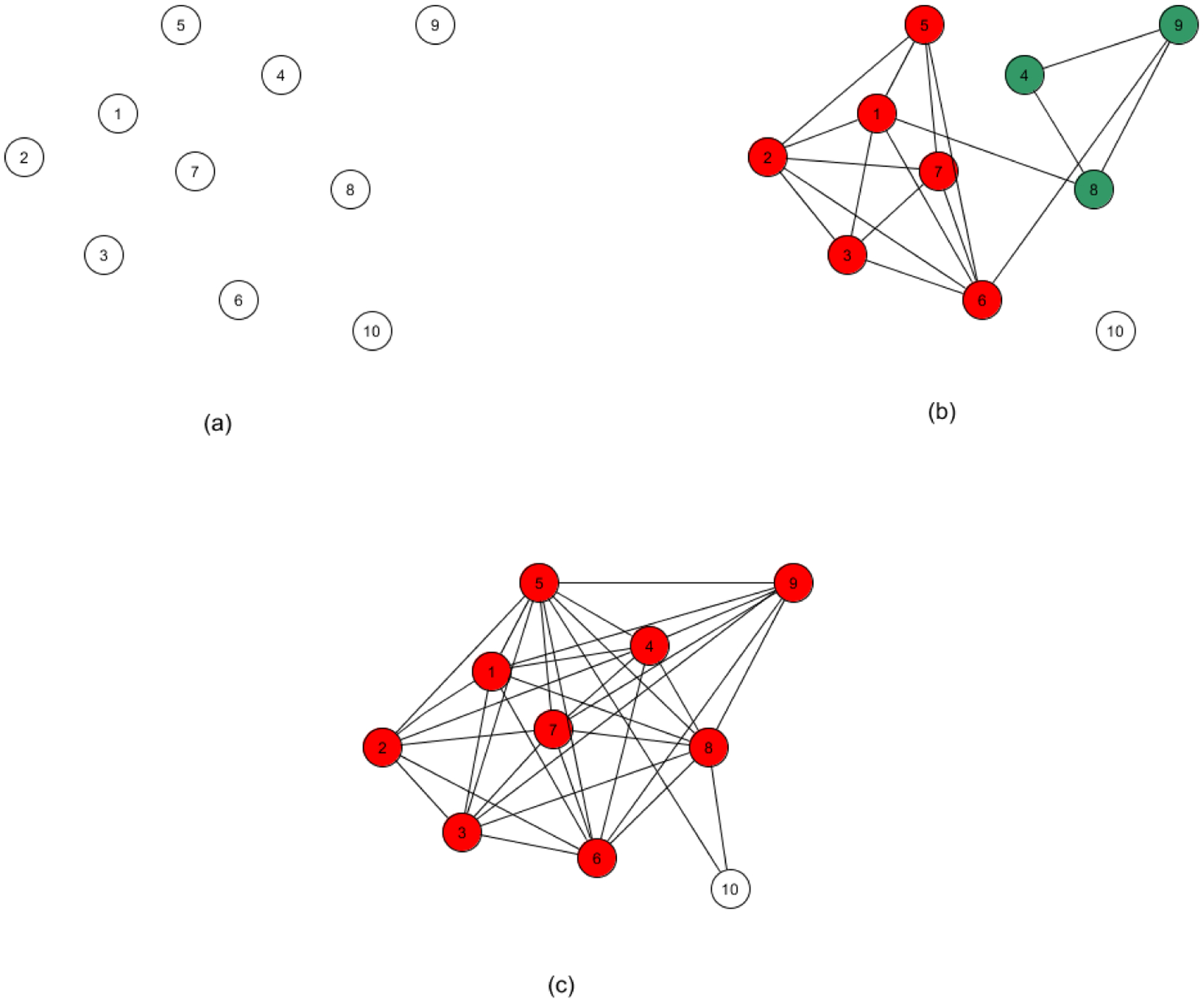
Outlier detection using paracliques. **(a)** A normalized threshold of 1.0 usually produces an empty graph. **(b)** As the threshold is lowered, more edges are added and paracliques begin to form and merge. **(c)** If a vertex consistently joins no paraclique, then it is flagged as a potential outlier.

The methodology just described is readily extensible to automating the task of outlier detection. This follows from the observation that an outlier would be expected to appear as its own distinct subtype, and not reside in a paraclique of even modest size. Although detection may be accomplished with our algorithms in several ways, we endorse the use of thresholding, as follows. A normalized threshold of 0.0 will of course produce a single large clique, and a threshold of 1.0 will generally yield an edgeless graph, under the assumption that no two samples are perfectly correlated. As the threshold value is lowered from 1.0, the effect on cliques and paracliques is slightly nuanced. As more and more edges are added, cliques and paracliques will get larger but also begin to merge. If a vertex consistently fails to join any of these dense subgraphs, then the sample it represents is flagged as a potential outlier. The process is illustrated in Figure 2. At this point, it may be tempting simply to single out isolated vertices, but at any given threshold a vertex may of course have a variety of neighbors and yet still be a member of no paraclique.

While this approach has intuitive appeal, we conducted a series of six experiments using known misclassifications to test its limitations. We formed test instances by introducing data from one randomly chosen healthy sample into data from the case samples for breast cancer (GSE10810), chronic lymphocytic leukemia (GSE8835), colorectal cancer (GSE9348), lung cancer (GSE7670), pancreatic cancer (GDS4102), and prostate cancer (GSE6919). For the breast, colorectal, lung, and pancreatic cancer sets, we observed that the normal sample was either the last or the next to last vertex to be drawn into the final paraclique. For the chronic lymphocytic leukemia and prostate cancer sets, we found instead that the healthy sample fell into a large paraclique early on and stayed there. From our previous experience with outlier detection [24], these observations suggest to us that although paraclique has a pronounced potential to serve as an automated outlier screening tool, feature selection [66] should probably first be performed to reduce any positive bias that results from whole genome correlations. We will revisit this topic in the next section.

## 5. Summary, Discussion and Directions for Future Research

We have developed and described a disease classification strategy based on the paraclique algorithm that can identify putative subtypes, segregating samples based on signatures in their molecular profiles. Although our tools are easily applicable to many types of biological data, we have focused on gene co-expression data largely thanks to their overall quality and availability. We have analyzed high throughput data taken from a dozen different disease samples obtained from the Gene Expression Omnibus and sought to validate the significance of our findings by reviewing the literature and examining ontological enrichment for the biological relevance of genes differentially expressed across putative subtypes. We also performed testing over data augmented with phenotypic information for known subtypes. Overall, the results of this study indicate a strong utility for this approach in the confirmation of known, and the discovery of novel, disease subtypes. Additionally, we described the extension of our methodology to the task of outlier identification. By iteratively lowering the threshold and re-running the paraclique algorithm, we can detect samples resistant to subtype coalescence. Such a finding can point to critical clinical errors such as tissue misclassifications and/or patient misdiagnoses. Throughout, our aim has been to employ scalable, cutting edge graph theoretical methods that can help automate the disease subtyping process, which can in turn accelerate the pace of discovery and lead to improvements in targeted therapies.

We emphasize that this exploratory effort has focused exclusively on unsupervised techniques and tools that require no prior knowledge. To keep things simple, we even refrained from fine tuning the glom term for each dataset. This, therefore, bolsters the argument that the methods we have espoused are really quite effective. In large-scale clinical applications, however, techniques such as feature selection and paraclique anchoring will almost surely prove helpful to narrow the focus on genes or other variables of interest and their disease-associated relationships. In the context of community-acquired pneumonia, for example, we have previously found it advantageous to anchor paraclique analytics at the interleukin genes *IL-6* and *IL-10*. See [29].

To place these results in proper context, we note that any subtyping method based on tissue morphology or molecular signatures is almost certain to be highly imperfect. In addition, this holds true whether it be implemented in silico or conducted manually by a human pathologist. A 2015 study [38] underscores this problem. There, an expert panel of pathologists created a baseline diagnosis based on consensus of opinion for 240 breast tissue biopsies with samples that included malignancy, pre-cancerous cells and benign tumors. Pathologists from eight states with at least one year of experience in diagnosing cancer were then invited to examine these samples. In total, 115 of them completed their analysis and provided their best diagnoses. Although findings showed that 96% of the invasive breast cancer samples had been diagnosed in concordance with expert consensus, 13% of the diagnoses underreported the severity of stage I breast cancer, while 48% (17%) underreported (overreported) the severity of precancerous samples. False negatives and false positives, such as these, can have devastating effects on patients. They may also lead to a wide spectrum of poor outcomes that include excessive delay, unnecessary treatment, additional expense, needless worry, and even premature morbidity and death.

Finally, we wish to emphasize that this work represents but a first step in determining the utility of paraclique in the molecular subtyping of disease. Although clique-based methods have been used as a basis for tasks such as biomarker detection and gene network elucidation, disease subtyping has received surprisingly little attention. In future work, it would thus be interesting to see systematic comparisons of this and other emergent sub-typing technologies. Numerous other research directions beckon. For example, we would like to gain a better understanding of the impacts of improved feature selection, and see extensions of the basic method to multiple heterogeneous data types, an area that has attracted a flurry of recent attention [67–69]. Collaborative opportunities to partner with disease specialists may of course also help in subtyping verification via graph theoretical methods at large. Better thresholding and filtering methods may be studied as well, in hopes of increasing the accuracy of subtyping and, in turn, reducing the likelihood of confounding factors. In conclusion, we observe that the overall approach we have described can be applied to numerous other sorts of biological data, as well as data from application domains as diverse as cyberattack detection and social network analysis.

## Supplementary Material

algorithms-14-00063-s001

## Figures and Tables

**Table 1.** Subtyping datasets. A profile of the datasets used in this study.

Disease	GEO Accession	Patients		Probes	
		Case	Control	Initial	Filtered
Asthma	GSE4302	42	28	54,675	2322
Breast Cancer	GSE10810	31	27	18,382	11,531
Chronic Lymphocytic Leukemia	GSE8835	24	12	22,283	1338
Colorectal Cancer	GSE9348	70	12	54,675	22,968
Lung Cancer	GSE7670	27	27	22,283	7458
Multiple Sclerosis	GDS3920	14	15	54,674	9844
Pancreatic Cancer	GDS4102	36	16	54,613	23,711
Parkinson’s Disease	GSE20141	10	8	54,674	6625
Prostate Cancer	GSE6919	61	63	12,625	1531
Psoriasis	GSE13355	58	58	54,675	29,407
Schizophrenia	GSE17612	28	23	54,675	4250
Type 2 Diabetes	GSE20966	10	10	61,294	93

**Table 2.** Subgroups identified. Summary of the numbers and sizes of putative subgroups identified by our methods in testing data.

Disease	Subgroups Identified	Subgroup Sizes
Asthma	3	31, 8, 3
Breast Cancer	2	22, 5
Chronic Lymphocytic Leukemia	2	4, 18
Colorectal Cancer	2	63, 5
Lung Cancer	2	21, 5
Multiple Sclerosis	2	11, 3
Pancreatic Cancer	2	31, 5
Parkinson’s Disease	1	8
Prostate Cancer	2	56, 3
Psoriasis	2	49, 5
Schizophrenia	2	19, 6
Type 2 Diabetes	1	9

**Table 3.** GO enrichments. Listed is the GO term category with the lowest enrichment *p*-value of the 100 most differentially expressed genes for each disease in this study.

Dataset	GO Category	*p*-Value
Asthma GSE4302	Oxireductase	1.1 × 10^−4^
Breast Cancer GSE10810	Secreted	1.0 × 10^−13^
Chronic Lymphocytic Leukemia GSE8835	Mhc ii	2.4 × 10^−15^
Colorectal Cancer GSE9348	Translational elongation	2.8 × 10^−28^
Lung Cancer GSE7670	Secreted	7.7 × 10^−10^
Multiple Sclerosis GDS3920	Translational elongation	1.9 × 10^−34^
Pancreatic Cancer GDS4102	Signal	4.59 × 10^−15^
Prostate Cancer GSE6919	Translational elongation	4.92 × 10^−46^
Psoriasis GSE13355	Immune response	3.5 × 10^−15^
Schizophrenia GSE17612	Organelle membrane	5.24 × 10^−4^

**Table 4.** Cluster compositions based on known subtypes. Shown is a breakdown of the subtypes obtained from datasets with best available ground truth for paraclique, k-means, and hierarchical clustering.

Paraclique Results						
Gastric Cancer			NSCLC			
	Paraclique Sizes			Paraclique Sizes		
	29	16		26	12	8
Subtype			Subtype			
proliferative	1	12	AC	23	0	8
invasive	19	1	SCC	3	12	0
metabolic	9	3				

k-Means Results					
Gastric Cancer			NSCLC		
	Cluster Sizes			Cluster Sizes	
	26	44		28	30
Subtype			Subtype		
proliferative	0	29	AC	18	22
invasive	25	1	SCC	10	8
metabolic	1	14			
Hierarchical Clustering Results					
Gastric Cancer			NSCLC		
	Cluster Sizes			Cluster Sizes	
	33	37		9	49
Subtype			Subtype		
proliferative	0	29	AC	0	40
invasive	22	4	SCC	9	9
metabolic	11	4			

## Data Availability

All data used in this study is available for public download from the Gene Expression Omnibus (GEO) website at https://www.ncbi.nlm.nih.gov/geo/ (accessed on 15 October 2020).
